# Traumatic insemination is not the case in three *Orius* species (Heteroptera: Anthocoridae)

**DOI:** 10.1371/journal.pone.0206225

**Published:** 2018-12-05

**Authors:** Kiyoko Taniai, Toru Arakawa, Taro Maeda

**Affiliations:** Division of Insect Sciences, The National Agriculture and Food Research Organization (NARO), Tsukuba, Japan; USDA Agricultural Research Service, UNITED STATES

## Abstract

Traumatic insemination (TI) is an extraordinary style of mating behavior wherein the female integument is pierced by the male extragenital structure to transfer the spermatozoa into the female’s body through wounding. Flower bugs of the genus *Orius* belong to the family Anthocoridae (Heteroptera), which is referred to as the “TI family”. Males possess sharp shaped extragenitalia, and females receive the extragenitalia using the copulatory tubes, which are specialized extragenital structures in *Orius* species. Since TI is not well studied in insects possessing the copulatory tube, we examined the genital structures and copulatory processes of three species, *Orius strigicollis*, *O*. *sauteri*, and *O*. *minutus*. Scanning electron microscopic observations revealed the positions of male extragenital structures during copulation. A needle-like flagellum was deeply inserted into the female intersegment between the abdominal VII and VIII segments, while the curved part of a sickle-like cone forced the intersegment to expand. No scars were detected around the copulation region after copulation. The copulatory tube adhered to the interior of segment VII, and the interior integument around the copulatory tube remained intact after copulation. On the basis of these results, TI does not occur in these *Orius* species. A pair of seminal conceptacles, which exists in typical TI insects, was found at the base of the oviducts in *O*. *strigicollis*. The distal end of the copulatory tube connected to a closed bag with a double-membrane, termed the sperm pouch. The sperm pouch was filled with filamentous structures after copulation and structures with equivalent forms were observed in adult male testis. These structures, considered to be spermatozoa, persisted in the pouch for at least two weeks after copulation, suggesting that the pouch is a long-term spermatozoa storage organ.

## Introduction

Traumatic insemination (TI) has been widely observed in the animal kingdom in nematodes, arthropods, flatworms, rotifers, snails, slugs, and amphibians [1, 2]. TI has independently evolved in at least 36 taxa [2] and occurs in various forms, which can be categorized by the site of wounding, the device (organ) used in TI, the materials transferred through the wounding, and female-male co-evolution [1]. TI is generally costly for the wound recipient because of physical damage and the risk of infection [3, 4]. The significance of TI is thought to be to anchor the female during copulation, and/or to offer a competitive advantage to the male, and/or to stimulate reproduction, and/or to increase the fertilization rate [1].

In insects, TI occurs in multiple orders, but most of the TI insects belong to the infraorder Cimicomorpha (Heteroptera). Within the Cimicomorpha, TI has independently evolved at least three times in the families Cimicidae, Miridae, and Nabidae [5]. Tatarnic et al. [5] suggested that five of the six families (Plokiophilidae, Lyctocoridae, Anthocoridae, Cimicidae, and Polyctenucudae) in the Cimicoidea employ TI. Extensive studies on TI have been conducted using bed bugs from the Cimicidae [6, 7, 8]. The extragenital structure, called the ectospermalege, is located at the fourth sternum in the female body, and spermatozoa are received by a special endodermal pouch, called the mesospermalege located underneath the ectospermalege. The spermatheca is absent in these insects, instead a pair of seminal conceptacles or pseudospermatheca at the base of the lateral oviducts receives the spermatozoa, which migrates from the mesospermalege in the hemolymph. It has been reported that members of the tribe Xylocorini (Anthocoridae) practice TI, where insemination is carried out hemocoetically [6, 9]. Unique structures in the copulation organs exist in the family Anthocoridae. Extragenital insemination (EI), in which copulation occurs outside the female reproductive tract, is the common mode of copulation among the Anthocoridae [10]. Females of the four tribes of Anthocoridae (Anthocorini, Blaptostethini, Oriini, and Scolopini) possess a specialized extragenital structure termed the copulatory tube, which is located in the intersegmental membrane between abdominal segments VII and VIII [11]. A copulatory tube is an extragenital structure in TI insects [12]. It is also speculated that members of the genus *Orius* practice TI based on the blade-like structure of the male intromittent organ [2], however, TI using the copulatory tube has to date not been well elucidated.

A number of species from the Anthocoridae family have been used as effective biopesticides worldwide [13]. Members of the genus *Orius* (Oriini, Anthocoridae) are predators of small-sized arthropod pests such as thrips, ticks, spider mites and aphids. The three species (*O*. *sauteri*, *O*. *strigicollis* and *O*. *minutus*) are promising biological control agents for vegetable pests in Japan [14–17]. Among these three species, only *O*. *strigicollis* is commercially available (since 2001) as a biological control agent, and is used in greenhouse-grown vegetables to control thrips. The natural habitat of *O*. *strigicollis* is limited to the southwest coast of Japan; however, mass-produced *O*. *strigicollis* might expand its habitat to that of its indigenous natural enemies, *O*. *sauteri* and *O*. *minutus*. Since these three species resemble each other, it is possible that inter-specific crossing occurs. When dealing with such phenomena, it would be beneficial to understand the mating systems of these insects.

In this study, we examined the genital structures and copulatory processes of three *Orius* species. We aimed to assess whether TI occurs in these three *Orius* species, and the function of two major parts of the male extragenitalia (the flagellum and the cone) during copulation. The results revealed that the copulation processes of all three *Orius* species are not associated with wounding; therefore, we concluded that TI does not occur in the three *Orius* species studied.

## Materials and methods

### Insects

*Orius sauteri* (Poppius) was collected from red clover, while *O*. *minutus* (L.) was collected from chestnut plants in Ibaraki Prefecture, Japan. *O*. *strigicollis* (Poppius) was purchased from Arysta LifeScience Corpo (Tokyo, Japan). These three species were maintained in different rooms to avoid mixing. Rearing conditions were the same for all three species. Approximately 30 pairs were kept in a plastic box (24 × 17 × 5 cm) at 25 ± 1 °C, 60–70% RH, under a 16hL: 8hD photoperiod. Eggs of the mill moth (*Ephestia kuehniella*) (Ga-Ran; Agrisect Inc., Ibaraki, Japan), the Mexican stonecrop (*Sedum mexicanum*), and a tube of water with a cotton cap were supplied twice a week. The fifth-instar larvae were collected and individually reared in plastic tubes (1 cm diameter × 7.5 cm length). One week after eclosion, a virgin female and a virgin male were placed together in a plastic tube, the couple was allowed to mate, and the female was then separated from the male and maintained individually. A designated mated female was a female that laid eggs three days after copulation. A female designated as a virgin female continued to be reared individually for a similar period after eclosion.

### Scanning electron microscopy

Scanning electron microscopy (SEM) was used to observe male genitalia, the position of male genitalia during copulation, and virgin and mated females for possible abdominal surface scars. Whole male bodies and copulating pairs were frozen in liquid nitrogen and kept overnight at -80 °C. After removal from -80 °C, the sample was stuck to an aluminum plate, and stored at room temperature under 50–70% humidity for 24 h. The sample was then placed in a desiccator chamber for 3–4 days under 11% humidity with saturated lithium chloride solution. Once completely dried, the sample was sputter-coated with gold using an SC-701 Quick Coater (Sanyu Denshi, Tokyo, Japan). Several copulating pairs of *O*. *strigicollis* were slightly separated by carefully using the thinnest insect pin (Shiga Insect Promotion Company, Tokyo, Japan) right before coating. This partial separation of mated pairs was successful only with *O*. *strigicollis*, pairs of the other two species were easily separated with minimal force. In these cases, only the separated male was coated with gold to observe the extragenitalia. Two males, two couples, and two partially separated pairs or two separated males were observed for each species.

To observe the abdomen, a female was anesthetized on ice, and the abdomen was removed, then placed in 50% ethanol. The abdomen was washed three times with 50% acetone, further washed twice with PBS, and fixed with 2.5% glutaraldehyde at room temperature for 2 h. After washing with PBS, the abdomen was dehydrated in an ethanol series. After dehydration with ethanol, the ethanol was replaced with *tert*-Butyl alcohol and the sample was frozen (solidify) at 5 °C in a minimal amount of *tert*-Butyl alcohol. This solution was freeze-dried using a freeze-drying machine (JFD-380P, JEOL, Tokyo, Japan). The next day, the dried sample was attached to an aluminum plate and sputter-coated with platinum. Abdomens of at least three virgin females and six mated females for each species were observed. Observations were conducted using a JSM-6301F scanning electron microscope (JEOL) and digital images of the samples were acquired using a software package (SEM Afore; JEOL).

### Stereoscopic and light microscopy

A stereoscopic microscope was used to observe male genitalia and the female sperm pouch. The male body was frozen in liquid nitrogen and then soaked in 20% KOH phosphate buffered saline (PBS, pH 7.5). The sample was stored at 5 °C for several days until the integument of the pygophore was sufficiently cleared to observe inside the genitalia, then washed with PBS. At least three males of each species were observed. In order to observe male genitalia during copulation, we have first tried to clear whole bodies of copulating pairs, however, in most cases the pairs immediately separated in the clearing solution. Thus, such separated males were observed for genitalia that had been rotated during copulation. To observe the sperm pouch, a virgin or a mated female of *O*. *strigicollis* was dissected in water and the abdominal segment VII was removed along with the copulatory tube and the sperm pouch. At least three samples were observed for virgin and mated females, respectively. All the samples were placed on the bottom of a 3.5 cm dish with several drops of water and observed under a stereoscopic microscope (MZ 16F, Leica Microsystems, Germany). Digital images of the samples were acquired using a VB7000 cooled CCD camera (KEYENCE, Osaka, Japan).

A light microscope was used to observe inside the female abdominal VII integument of the three species, and the reproductive organs of the *O*. *strigicollis* female. The female was dissected in water to remove the integument surrounding the abdominal segment VII, the integument was placed on a glass slide with the hemolymph-facing side placed up, then covered with a coverslip. For each of the three species, at least five virgin and five mated females were observed. To observe the positional relationship between the reproductive organs and the copulatory tube, the internal reproductive organs were removed from *O*. *strigicollis* females. As the reproductive organs were white or colorless, these organs were visualized by staining with 0.1% Toluidine Blue solution (pH 7.0) for a few seconds, then washed with PBS. Samples were placed on a glass slide and covered with a coverslip. Complete sets of reproductive organs were successfully removed from only one virgin and a few mated females. The samples were observed under a light microscope (Axio plan2, Carl Zeiss, Germany), and digital images were obtained using an AxioVision System (Carl Zeiss).

### Nuclear staining

In order to identify structures in the sperm pouch of copulated females, nuclear staining was performed with a slight modification of methods described by Sekine et al [18]. Briefly, the sperm pouch of virgin and mated females, and the male testis were removed and placed on a glass slide. The samples were washed twice with water, and fixed with 45% acetic acid for 1 min, and stained with a lacto-aceto-orcein solution (lactic acid:acetic orcein [3% orcein + 60% acetic acid] = 1:1) for 3 min. The samples were treated with 60% acetic acid for 30 sec, then a few drops of the lactic-acetic-orcein solution were added, and the samples were crushed with a coverslip. As the structures inside of the sperm pouch were not stained well, the coverslip was carefully removed and these samples were stained again with a few drops of the lacto-aceto-orcein solution for more than 5 min. The samples were covered with new coverslips and observed under a light microscope (Axio plan2). The sperm pouch from two virgin females, three females 4 h after copulation, three females two weeks after copulation, and testis from two 12-day-old males were used for nuclear staining observations.

## Results

### Male genitalia

**Fig 1:**
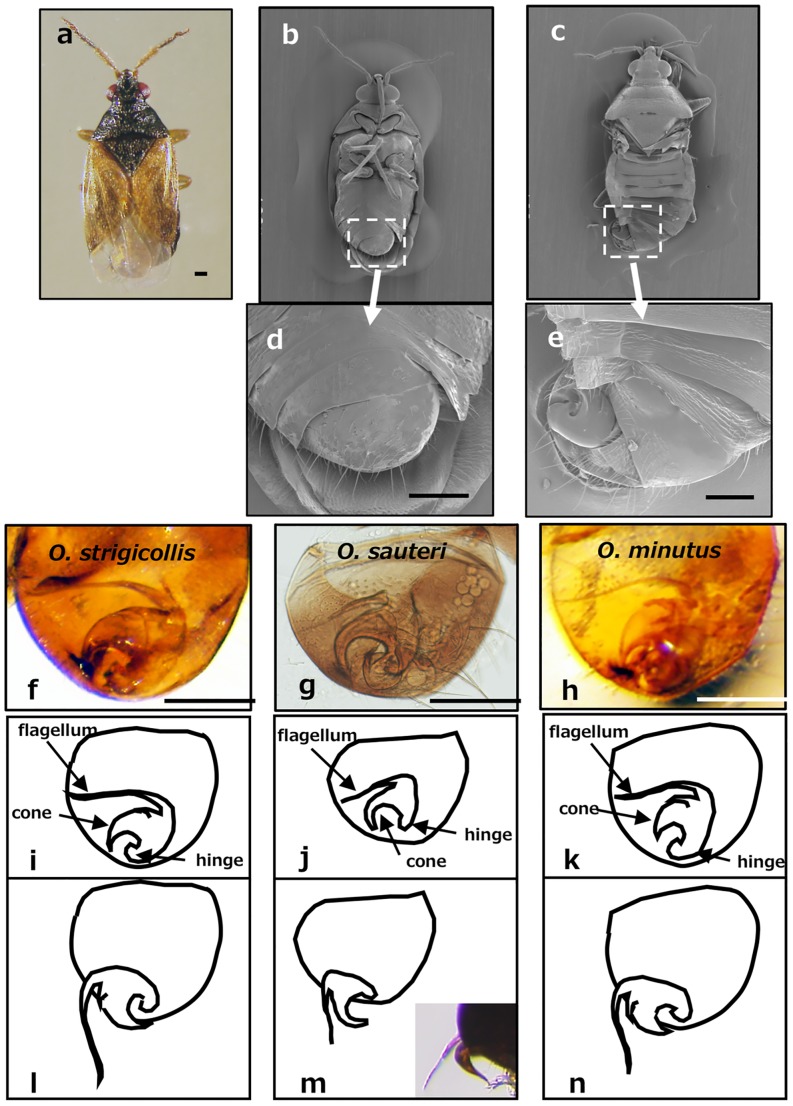
Male genital structure. Whole male *O*. *strigicollis* body viewed from the back (a) and SEM photos of the ventral side (b) and dorsal side without wings (c). Pygophores marked by the white squares with dotted lines are shown at higher magnification (d, e). Comparison of genital structures of the three *Orius* species; Ventral view of the pygophores after clearing with KOH (f, g, h); Schematic illustrations of the genitalia in normal positions (i, j, k) and rotated positions during copulation (l, m, n). A photograph of the rotated *O*. *sauteri*, which is largely exposed on the outside is incorporated in Panel m. Scale bars in Panels a and d-h indicate 100 μm.

The three *Orius* species are similar in appearance; a representative *O*. *strigicollis* body is shown in Fig 1a. Abdominal segment IX of the male (pygophore) was twisted clockwise 180 ° by nature (Fig 1b and 1c, S1 Fig) and the extra genitalia, which are normally stowed under the wings, are oriented upward on the backside (Fig 1c, S1 Fig).

The pygophore has been enlarged in the photos below Fig 1b and 1c. Although male genitalia of all the three species consisted of a needle-like flagellum and a sickle-like cone, parts of these structures differed slightly from one another, which can be used for classification. The flagella of *O*. *strigicollis* and *O*. *minutus* were long and curved like a whip (Fig 1d and 1f), while the flagellum was almost straight in *O*. *sauteri* (Fig 1e). There was a spinous protrusion on the side of the cone in *O*. *strigicollis* and *O*. *minutus*, and the size of the protrusion was larger in *O*. *minutus* than in *O*. *strigicollis* (Fig 1d and 1f). Since it was difficult to see dark-colored organs against the background of the dark-hued bodies, rotated genitalia of the three species are illustrated in Fig 1l–1n.

### Male genitalia position during copulation

**Fig 2:**
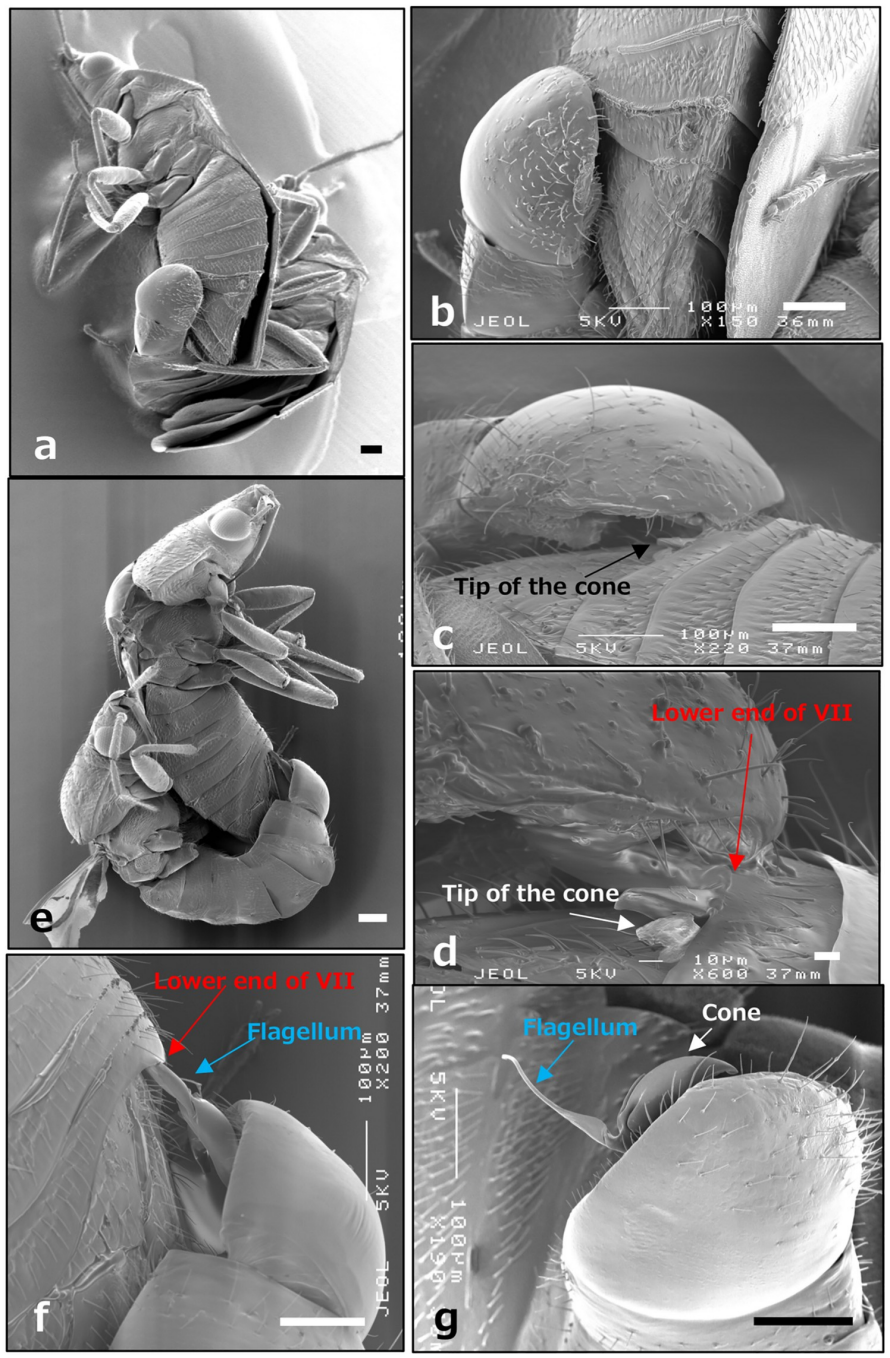
Male genitalia position during copulation. SEM observation of female and male *O*. *strigicollis* during copulation (40× in Panel a and 150× in Panel b). The bodies were coated with gold and the pygophore was manually separated from the female (220× in Panel c and 600× in Panel d). The pygophore was further separated and observed from the opposite side of that in Panel a (45× in Panel e and 200× in Panel f). The pygophore of *O*. *minutus* was completely detached from the female (Panel g). Scale bars at right bottom in all panels indicate 100 μm except for Panel d (10 μm).

The male captured the female from behind, and bent his abdomen toward the female abdomen, with the pygophore in tight contact with the middle of the female abdomen near the VII and VIII segments (Fig 2a and 2b, S2 Fig).

When the pygophore was slightly separated from the female, we observed that the tip of the cone protruded from under segment VII, but that a majority of the cone remained inserted under segment VII (Fig 2c). Based on orientation of the tip of the cone, it is unlikely that the cone pierces the female integument. Rather, the cone likely expanded the space of the intersegment by inserting between the VII and VIII segments (Fig 2c and 2d). When the male body was further manually separated using an insect pin, the male flagellum could be observed. The flagellum was inserted deeply into the intersegment even though the pygophore was separated to a certain extent from the female (Fig 2e and 2f). The orientation and position of the cone and flagellum in *O*. *minutus* was similar to that of *O*. *strigicollis* as shown in Fig 2g.

### Are there any scars on the female?

**Fig 3:**
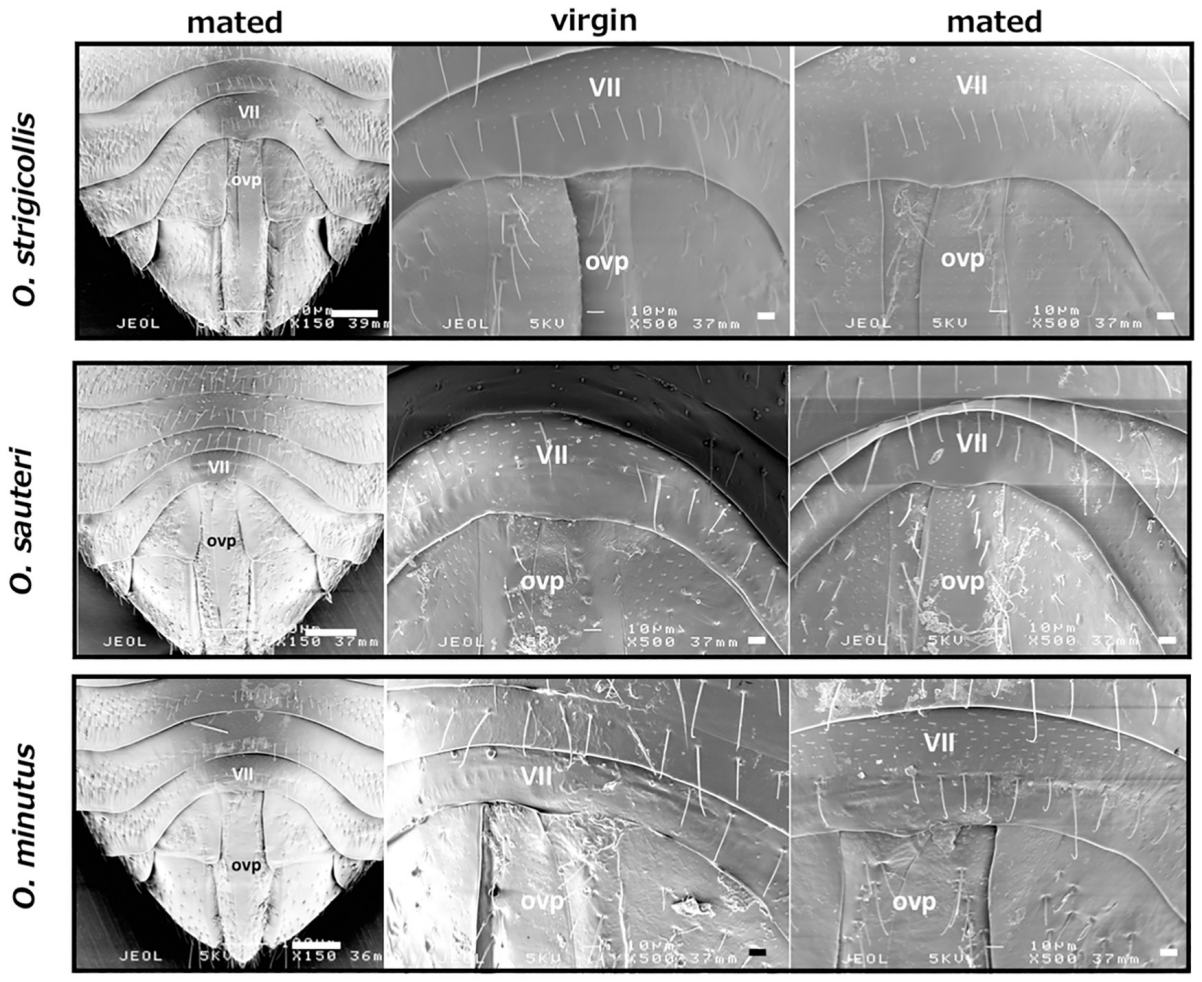
SEM observation of female integument. The left three photos of mated female abdomens were magnified 150×. Enlarged photos in the middle (virgin) and right (mated) were magnified 500×. VII, abdominal segment VII; ovp, the ovipositor at the center of segment VIII. Scale bars at bottom right in the 150× photos indicate 100 μm, and 10 μm in 500× photos.

SEM observation of a pair in copulation revealed that neither the cone nor the flagellum had pierced the female integument. To confirm if there were any scars on the female, we observed the abdominal surface using SEM. Abdominal segments V to VIII were observed at a magnification of 150×, and the area of pygophore contact (around the boundary of segments VII and VIII) was observed at a magnification of 500×. Although slight damage caused by sample preparation occurred in both virgin and copulated females, there were no apparent scars typical of copulated females detected in any of the three *Orius* species (Fig 3, S3 Fig).

**Fig 4:**
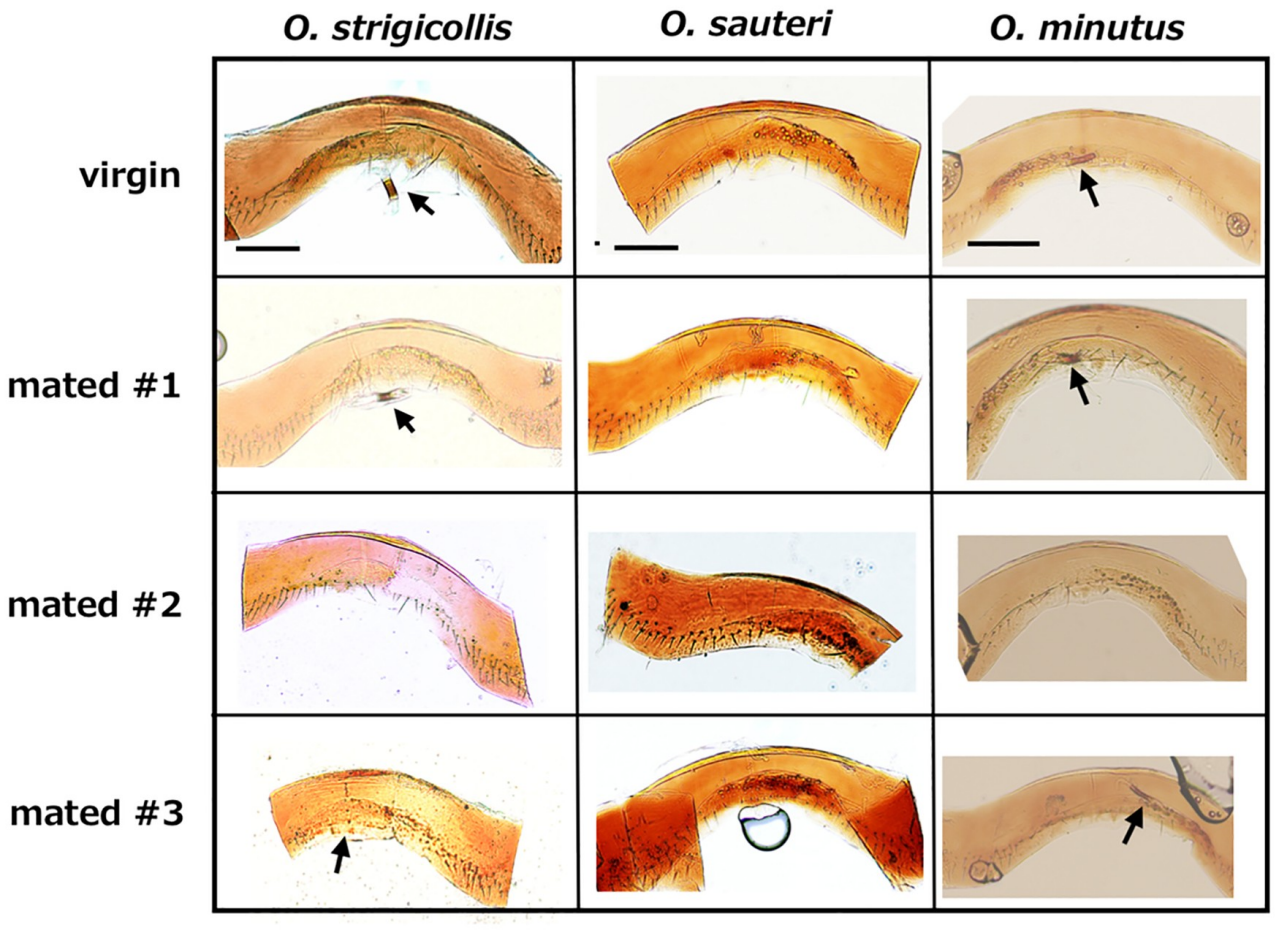
Interior side of female VII segment. One virgin and three mated female samples are indicated, respectively. Arrows indicate the copulatory tubes. Scale bars indicate 100 μm.

In order to examine whether the male scarred the interior side of the female integument, abdominal segment VII was isolated, and after clearing the integument, it was observed under a light microscope. The central portion of the segment was thinly arched (Fig 4).

The copulatory tube was occasionally attached to the thinner portion in *O*. *strigicollis* and *O*. *minutus* (Fig 4), while the copulatory tube of *O*. *sauteri* was very fragile and was lost during clearing. The copulatory tubes were cylindrical structures with some pigmented regions. Shapiro et al. [19] reported that the copulatory tubes were attached directly to the interior integument and that the opening positions were visible in *O*. *insidiosus* (Say) and *O*. *pumilio* (Champion); however, the opening of the copulatory tubes of the three species in the present study were not sclerotized and were located in the intersegmental membrane that lacked color. Therefore, it was quite difficult to find the opening site in the three species. The inserted area of the flagellum during copulation was almost in the middle of segment VII, however, no scars were detected in any of the three species (Fig 4) and we could not detect any differences between virgin and mated females.

### Sperm pouch as a probable storage organ of spermatozoa

**Fig 5:**
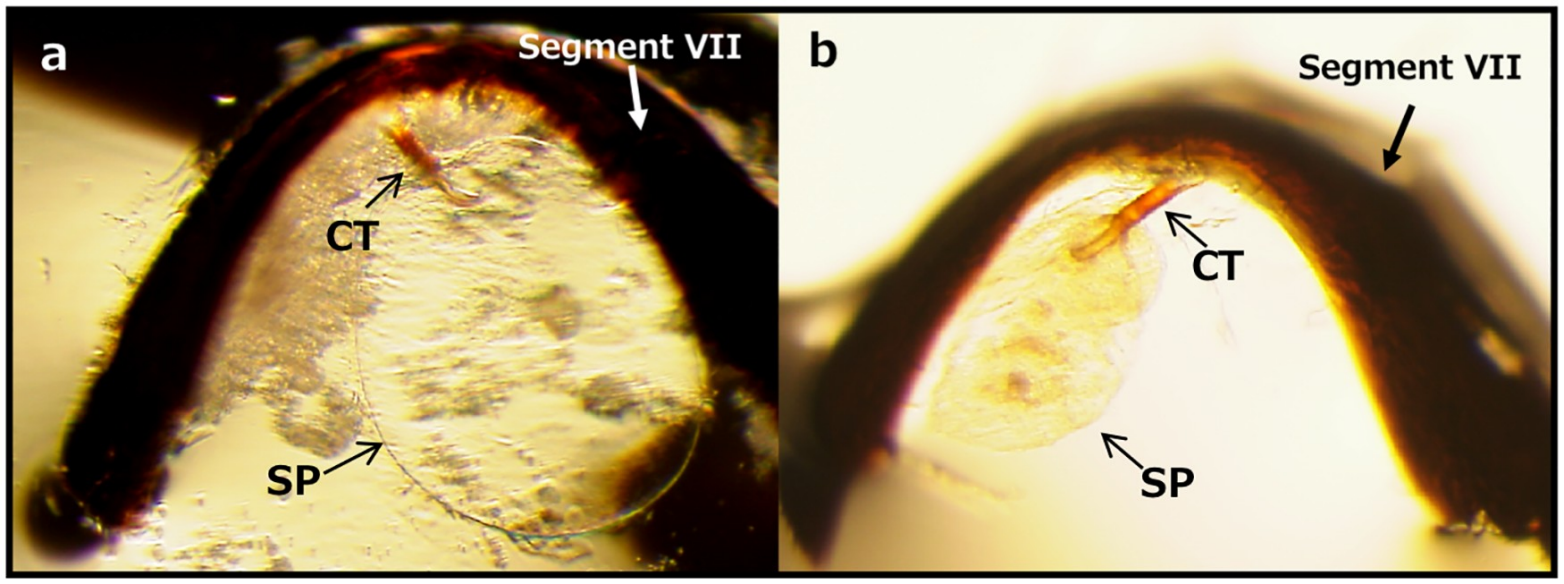
Copulatory tube with sperm pouch. A copulatory tube and sperm pouch isolated from a virgin female (a) and mated female (b) of *O*. *strigicollis*. The copulatory tube was connected to abdominal segment VII via an intersegmental membrane. CT, copulatory tube; SP, sperm pouch.

The sperm pouch is defined as the organ that corresponds to the mesospermalege in bed bugs, and its function is temporary storage of spermatozoa. The sperm pouches of the three *Orius* species were closed bags with double membranes. When a virgin *O*. *strigicollis* female was dissected in water, the sperm pouch filled with water and became almost transparent (Fig 5a), but the sperm pouches in mated females did not fill with water, but they were filled with muddy clumps (Fig 5b).

**Fig 6:**
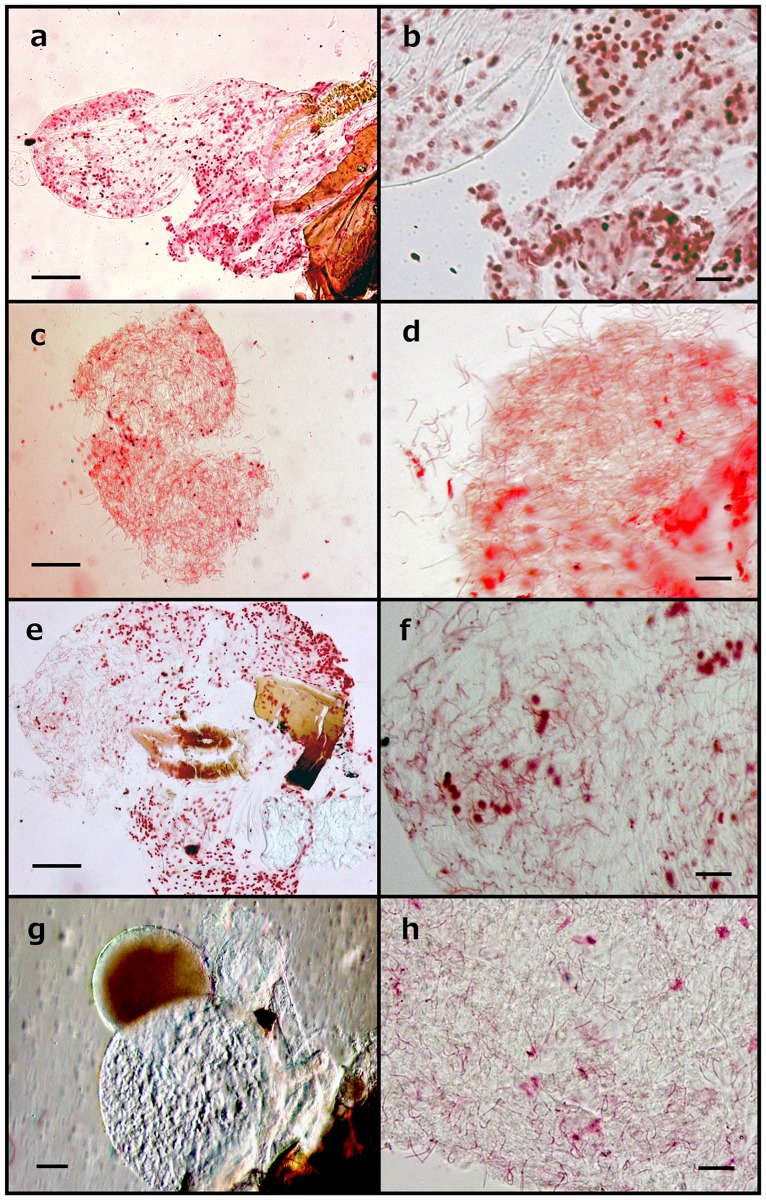
Filamentous structures in the sperm pouch. The sperm pouch was isolated from a *O*. *strigicollis* virgin female (a, b), from a female of 4 h after copulation (c, d), from a female 14 days (e, f). A male testis (g) and a stained testis (h). The large red circles in panels a-f are cell nuclei in the sperm pouch. The red filiform structures in panel c-f and h are most likely spermatozoa. Scale bars indicate 50 μm.

The muddy clumps in the sperm pouch were examined by nuclear staining. The filamentous structures could only be stained after crushing the pouch and re-staining, indicating that the staining solution was initially unable to penetrate into the inner membrane of the intact pouch. The sperm pouch of the *O*. *strigicollis* virgin female contained stained cell nuclei and no other structures were observed (Fig 6a and 6b).

The sperm pouch from females 4 h after copulation (Fig 6c and 6d) and 14 days after copulation (Fig 6e and 6f) were both filled with filamentous structures. These structures were equivalent in form to that of the testis (Fig 6h), suggesting they are spermatozoa.

### Reproductive tract with seminal conceptacles

**Fig 7:**
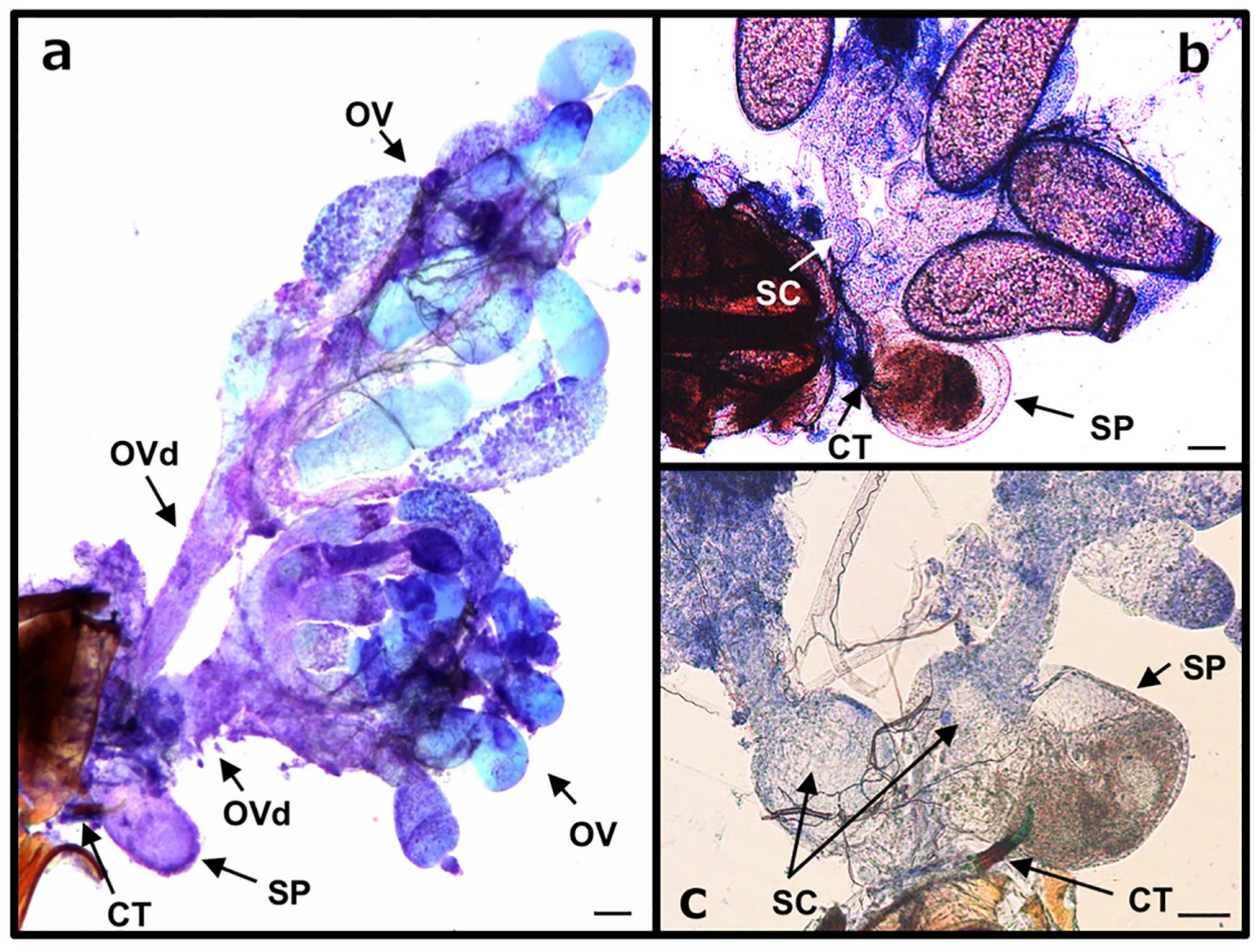
Reproductive organs of *O*. *strigicollis*. The ovary from a one-week-old virgin female (a), two-week-old mated female (b), and an approximately three-week-old mated female (c). The seminal conceptacles were hidden by the integument in Panel a. OV, ovary; OVd, oviduct; CT, copulatory tube; SP, sperm pouch; SC, seminal conceptacles. Scale bars in each panel indicate 100 μm.

The copulatory tube and surrounding structures were fragile and separated easily during dissection. These organs were relatively robust in *O*. *strigicollis* in comparison to the other two species, so we mainly used *O*. *strigicollis* for further experiments. We successfully dissected and removed the ovaries from virgin females, two-week-old mated females, and approximately three-week-old mated females (Fig 7).

**Fig 8:**
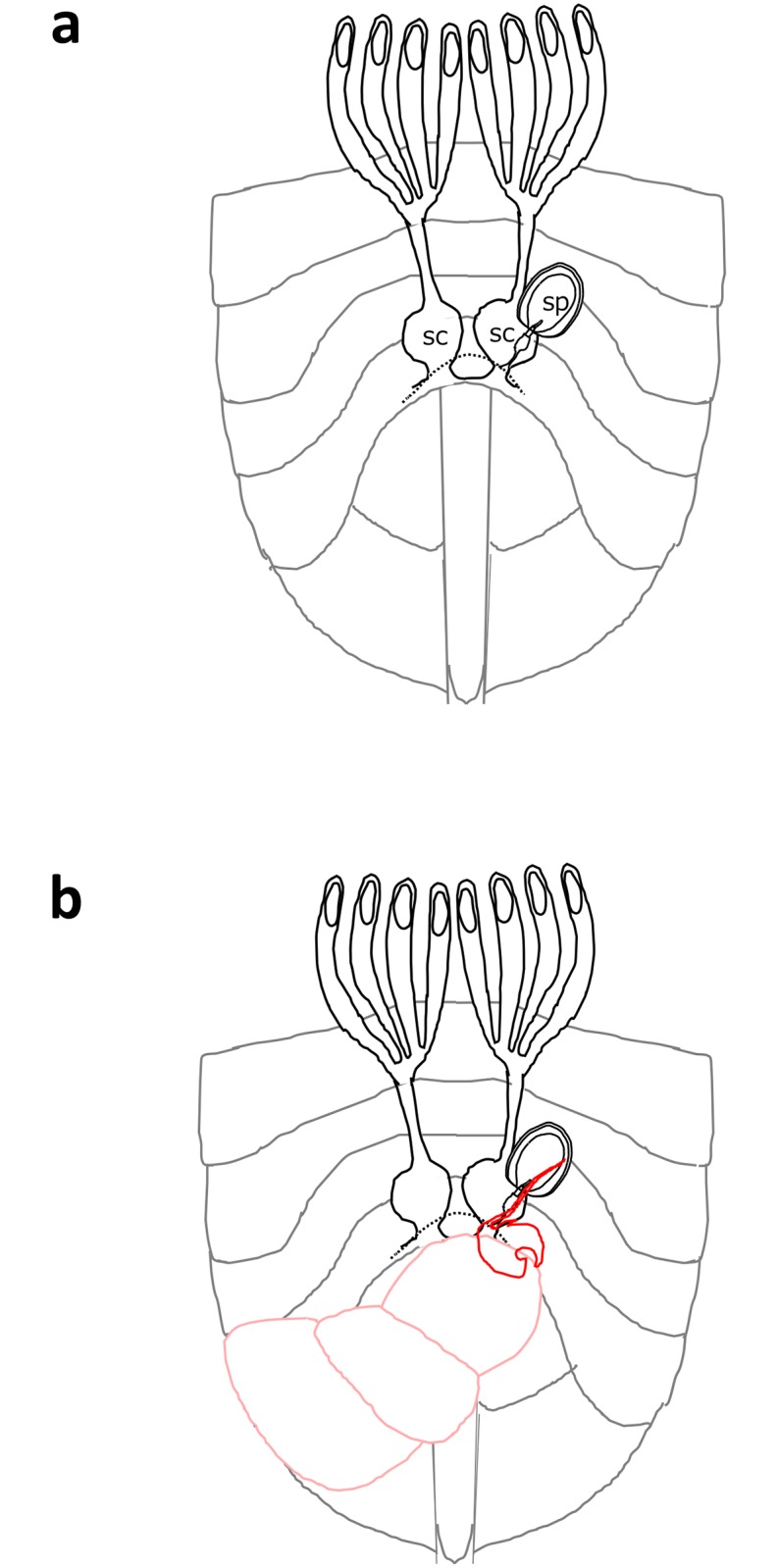
Female reproductive system. Schematic illustration of the ventral female abdomen (a). sp, sperm pouch; sc, seminal conceptacles. The pathway by which sperm traverse from sperm pouch to the seminal conceptacles is unknown. Schematic depicting positions of the male genitalia during copulation (b). The male abdomen and the extragenitalia are depicted by the red lines.

The ovaries and copulatory tube were removed together along with integument from the VII or VIII segment. Removal of the integument from the organs disrupted the structural relationship between the ovaries and the copulatory tubes. A pair of circular-shaped seminal conceptacles were found at the bottom of the oviducts of all female samples (Fig 7b and 7c). A sperm pouch was found at the distal end of the copulatory tube in all female samples (Fig 7a–7c). The sperm pouch and the reproductive organs are depicted in the schematic illustration shown in Fig 8a.

## Discussion

Reproductive systems and associated structures have uniquely evolved in some species, in particular those of the Cimicoidea superfamily. TI is widespread in the Cimicoidea; however, the term “TI” often seems to be confused with “EI”. TI is associated with the extragenital system in many species [6–7, 20–23]; however, the two phenomena are not the same. The reproductive structure of *O*. *strigicollis* consists of seminal conceptacles, suggesting that *Orius* and bed bugs are closely related. However, we found that the three *Orius* species in this study employed EI without wounding the female integument. On the basis of the observations in this study, we have concluded that TI does not occur in the three *Orius* species. The morphology of the male extra genitalia was blade-like in all three *Orius* species, however, a sickle-like cone was used as a spatula that was inserted into the female abdominal intersegment to force open a space for flagellum insertion. The needle-like flagellum was inserted intersegmentally between abdominal segments VII and VIII without damaging any thinner area of the integument. The copulatory tube was a partially sclerotized cylinder-like structure, which probably plays a role in supporting and guiding the flagellum into the sperm pouch. From these results, there is no expectation of damage to the female by TI during inter-specific crossing between the three species studied.

In insects that engage in EI, the sperm pouch is frequently considered a temporary storage appendage for spermatozoa [10]. However, in our study, the sperm pouch was filled with filamentous structures (which were presumably spermatozoa) even after depositing many eggs for more than 10 days, suggesting that the sperm pouch is a long-term spermatozoa storage organ. We observed that females of *O*. *strigicollis* are monandrous (unpublished data); therefore, spermatozoa may be stored in the sperm pouch for the female’s entire lifetime.

Among members of the Anthocoridae, only the Xylocorini have been reported to engage in TI. Since the infraorder Cimicomorpha has been proposed [24], classification of the Cimicomorpha including the Anthocoridae has not yet been established to date, despite extensive cladistic analyses performed by several groups [25]. Although the Xylocorini tribe is currently classified in the Anthocoridae [26], it has previously been included in other families (Cimicidae and Lyctocoridae) [10, 25]. Recent molecular phylogenetic analyses revealed that Xylocorini were excluded from the main Anthocoridae clade and were instead grouped with Cimicidae [27]. If Xylocorini belongs to a lineage different from the Anthocoridae, Anthocoridae should not be referred to as a TI family.

There are five tribes other than Xylocorini that belong to the Anthocoridae; Anthocorini, Blaptostethini, Cardiastethini, Scolopin, and Oriiini. Females in these tribes, with the exception of the Cardiastethini, have a copulatory tube. It is interesting to know whether insects in these tribes utilize TI or not. One possibility is that the copulatory tube has evolved in the Anthocoridae instead of developing TI. Another possibility is that TI evolved once in this clade, but it became obsolete in the Anthocoridae after the copulatory tube was gained. Since TI is a form of sexual conflict, females might have evolved the copulatory tube as a counter-adaptation to reduce physical damage. Males also could reduce the cost of strengthening the genitalia as a wounding device from loss of the TI behavior. In order to understand how TI evolved in the Anthocoridae, it is necessary to investigate the copulation processes and the morphology of reproductive organs in other members of this family.

## Supporting information

S1 Fig Male body. Whole body of *O*. *sauteri* (upper panels) and *O*. *minutus* (lower panels) viewed from the back (a), SEM photos of the ventral side (b) and dorsal side without wings (c). Pygophores marked by the white squares with dotted lines are shown at higher magnification (d, e). Scale bars in Panels d and e indicate 100 μm.
(TIFF)

S2 Fig Male genitalia position during copulation. SEM observation of female and male of *O*. *sauteri* (left panels) and *O*. *minutus* (right panels) during copulation (40× in Panels a, 150× in Panels b, 170× in Panel c of *O*. *sauteri* and 200× in Panel c of *O*. *minutus*). Scale bars in Panels b, c indicate 100 μm. The pygophore was slightly detached from *O*. *sauteri* female and completely detached from *O*. *minutus* female (each panel c). Red arrowheads indicate the cone of male genitalia.
(TIFF)

S3 Fig SEM observation of female integument. Mated female abdomens magnified 500×. VII, abdominal segment VII; ovp, the ovipositor at the center of segment VIII. Scale bars at bottom left in the panels “mated #2” indicate 10 μm.
(TIFF)
