# Supplementary figures and images for: Traumatic insemination is not the case in three Orius species (Heteroptera: Anthocoridae)
Source: PLoS One. 2018 Dec 5;13(12):e0206225. doi: 10.1371/journal.pone.0206225 (PMC6281218; doi:10.1371/journal.pone.0206225)

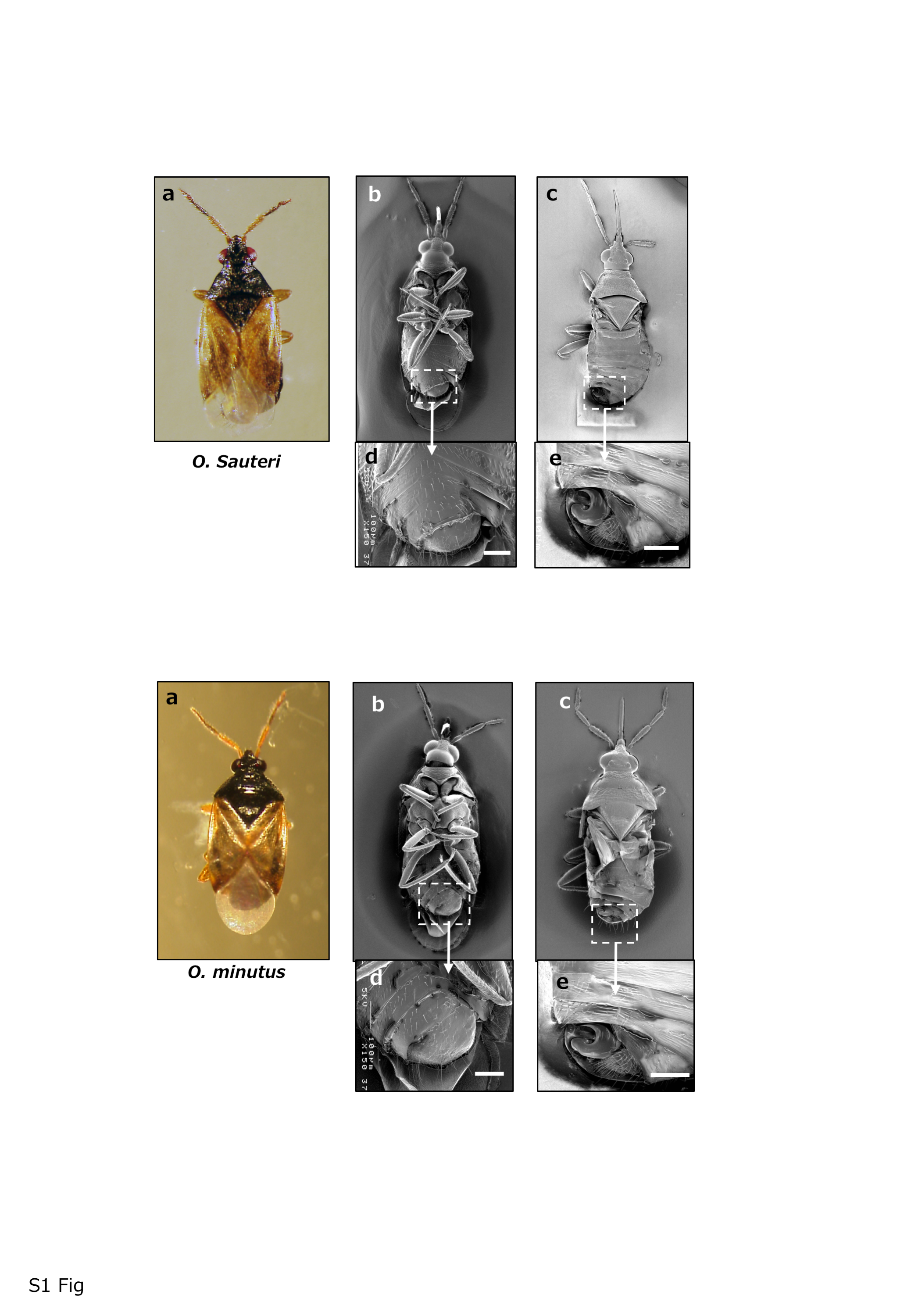

Supplement: S1 Fig — Whole body of O. sauteri (upper panels) and O. minutus (lower panels) viewed from the back (a), SEM photos of the ventral side (b) and dorsal side without wings (c). Pygophores marked by the white squares with dotted lines are shown at higher magnification (d, e). Scale bars in Panels d and e indicate 100 μm. (TIFF) [file pone.0206225.s001.tiff]

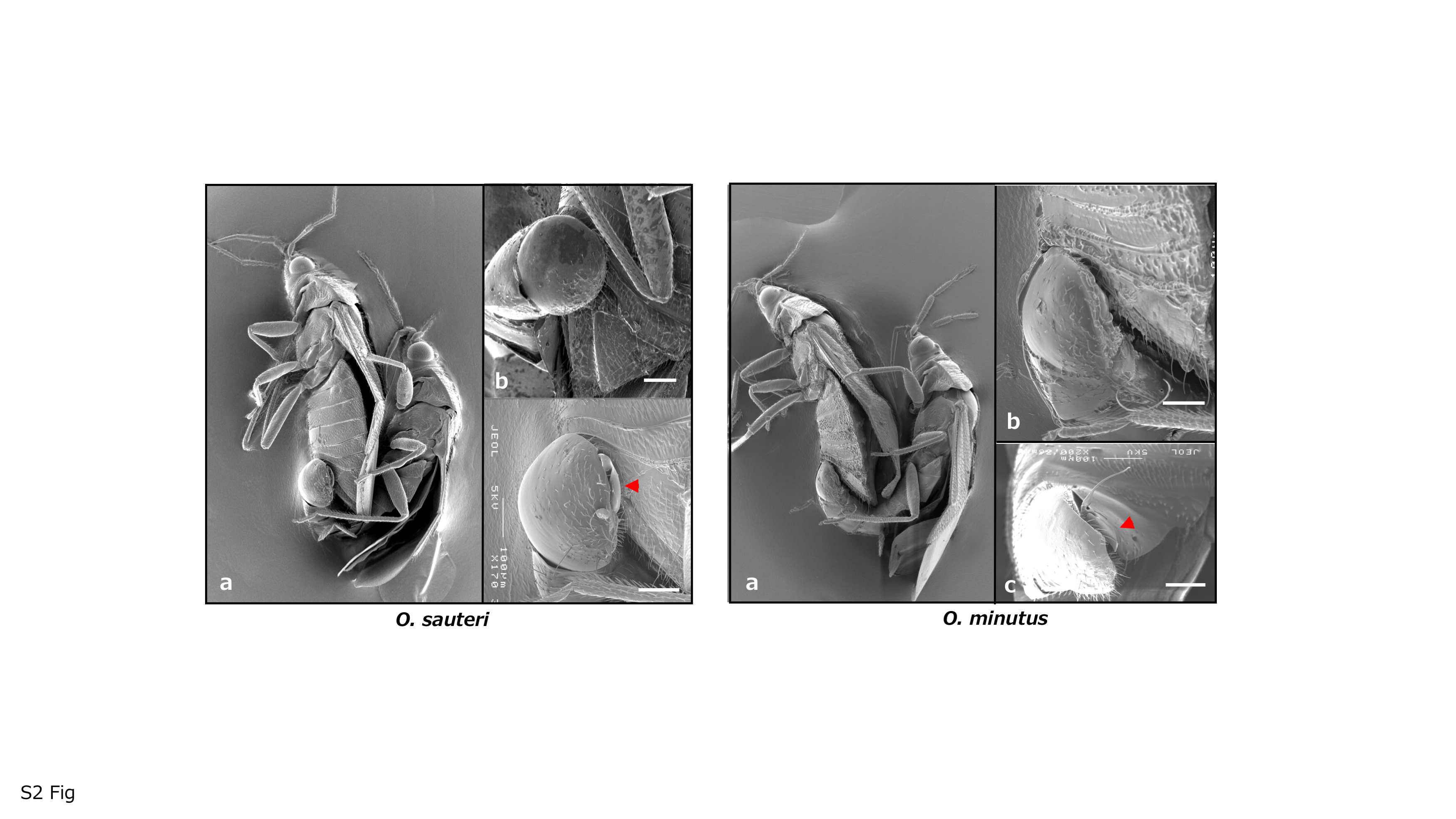

Supplement: S2 Fig — SEM observation of female and male of O. sauteri (left panels) and O. minutus (right panels) during copulation (40× in Panels a, 150× in Panels b, 170× in Panel c of O. sauteri and 200× in Panel c of O. minutus). Scale bars in Panels b, c indicate 100 μm. The pygophore was slightly detached from O. sauteri female and completely detached from O. minutus female (each panel c). Red arrowheads indicate the cone of male genitalia. (TIFF) [file pone.0206225.s002.tiff]

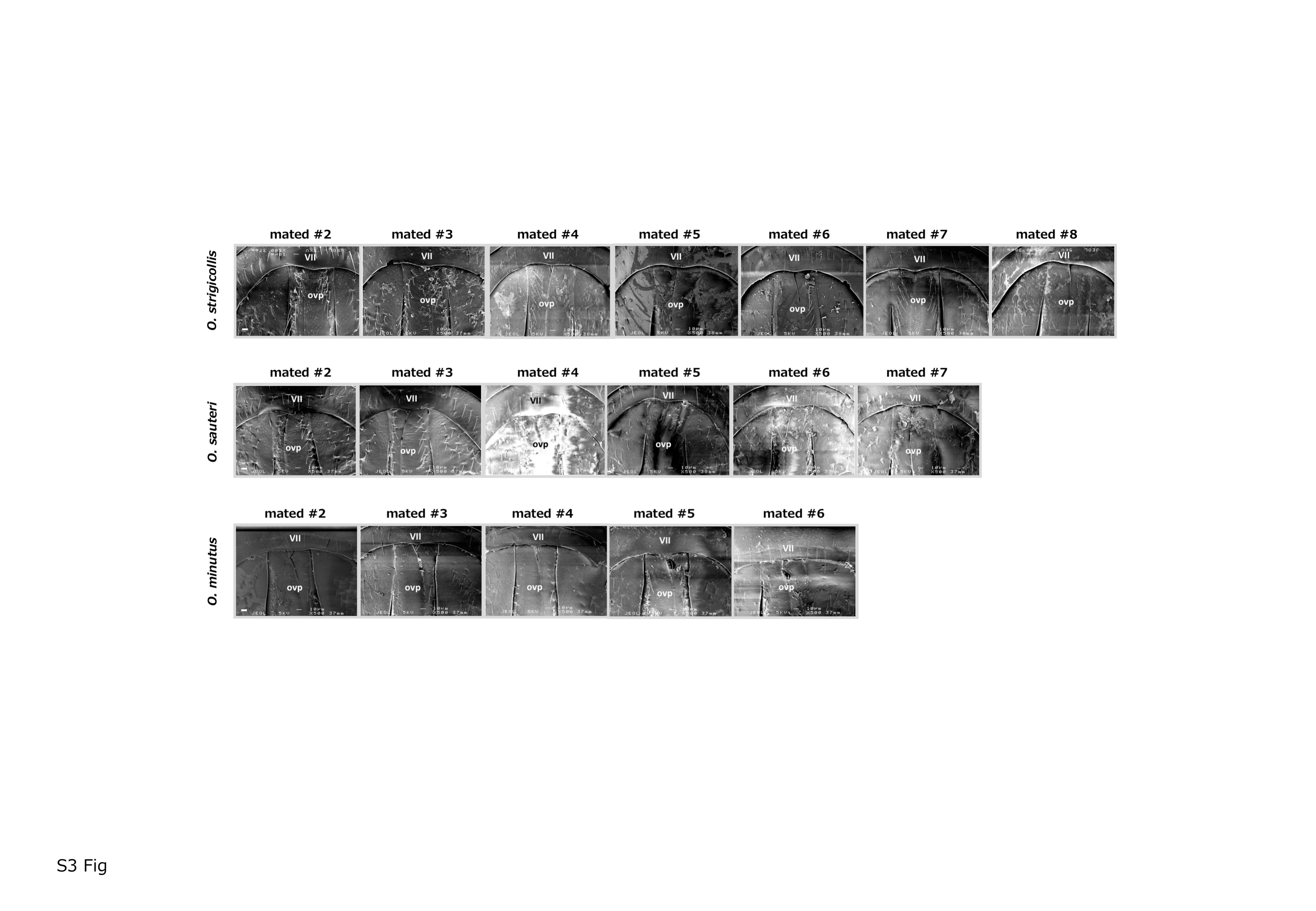

Supplement: S3 Fig — Mated female abdomens magnified 500×. VII, abdominal segment VII; ovp, the ovipositor at the center of segment VIII. Scale bars at bottom left in the panels “mated #2” indicate 10 μm. (TIFF) [file pone.0206225.s003.tiff]
